# A Modified Magnified Analysis of Proteome (MAP) Method for Super-Resolution Cell Imaging that Retains Fluorescence

**DOI:** 10.1038/s41598-020-61156-2

**Published:** 2020-03-06

**Authors:** Jiwon Woo, Jeong-Min Seo, Mirae Lee, Juyoung Kim, Sol Min, Sang-Tae Kim, Seockmo Ku, Jeong-Yoon Park

**Affiliations:** 1 The Spine and Spinal Cord Institute, Department of Neurosurgery, Gangnam Severance Hospital, Yonsei University College of Medicine, Seoul, 06273 Republic of Korea; 2 Brain Korea 21 PLUS Project for Medical Science, Yonsei University, Seoul, 03722 Republic of Korea; 3 Cellular Reprogramming and Embryo Biotechnology Laboratory and Dental Research Institute, Seoul National University School of Dentistry, Seoul, 08826 Republic of Korea; 4 Biomedical Research Institute, NeoRegen Biotech Co., Ltd., Gyeonggi-do, 16614 Republic of Korea; 5 Department of Neurology, Seoul National University Bundang Hospital, Seongnam-si, Gyeonggi-do 13605 Republic of Korea; 6 Fermentation Science Program, School of Agriculture, College of Basic and Applied Sciences, Middle Tennessee State University, Murfreesboro, TN 37132 USA

**Keywords:** Fluorescence imaging, Biomaterials - cells

## Abstract

Biological systems consist of a variety of distinct cell types that form functional networks. Super-resolution imaging of individual cells is required for better understanding of these complex systems. Direct visualization of 3D subcellular and nano-scale structures in cells is helpful for the interpretation of biological interactions and system-level responses. Here we introduce a modified magnified analysis of proteome (MAP) method for cell super-resolution imaging (Cell-MAP) which preserves cell fluorescence. Cell-MAP expands cells more than four-fold while preserving their overall architecture and three-dimensional proteome organization after hydrogel embedding. In addition, Optimized-Cell-MAP completely preserves fluorescence and successfully allows for the observation of tagged small molecular probes containing peptides and microRNAs. Optimized-Cell-MAP further successfully applies to the study of structural characteristics and the identification of small molecules and organelles in mammalian cells. These results may give rise to many other applications related to the structural and molecular analysis of smaller assembled biological systems.

## Introduction

Biological systems are stunningly complex, often consisting of millions of individual cells. Each cell can be classified as belonging to one of a number of distinct cell types, and is part of one or more closely interconnected functional networks. Cells are the basic structures of all living things, and individual cells often show clear heterogenicity. Studies using advanced techniques of subcellular and nano-scale imaging are essential for understanding the individual characteristics of cells. Over the past few decades, cell imaging analysis systems have been developed to observe cells in three-dimensions due to technological advances in confocal microscopy. However, further resolution via current imaging analysis through confocal microscopy is technically limited due to lens magnification, point spread function, and diffraction limitations^1–3^. It is therefore necessary to develop new technologies for single cell imaging that are capable of super-resolution.

Two approaches have been developed for the super-resolution imaging of cells. One approach includes super-resolution optical techniques, such as photoactivated localization microscopy (PALM), stochastic optical reconstruction microscopy (STORM), and stimulated emission depletion microscopy (STED)^4–6^. The other approach is super-resolution imaging by physical tissue expansion, including expansion microscopy (ExM), and magnified analysis of the proteome (MAP)^7,8^. Both super-resolution microscopy and tissue expansion techniques have advantages and disadvantages. Super-resolution microscopy can be applied to living cells, but is not easily applied, as the equipment is very expensive and cannot be used with thick tissue and conventional immunostaining^9^. In contrast, tissue expansion techniques may not be applied to living cells, but are usually less expensive and can be used on thick tissues^7,8^ *Chen et al*. have demonstrated that protease digestion of a hydrogel-tissue hybrid homogenizes the tissue’s mechanical characteristics, and allows approximately fourfold linear expansion. The technique was named, “expansion microscopy” (ExM)^7^. Although the use of ExM with conventional antibody and fluorescent proteins has been suggested recently, the protease digestion step in ExM causes a loss of proteins, which limits the number of protein structures that can be imaged in the same sample, and only small and thin tissues can be visualized^7,10^. To overcome the obstacles inherent to ExM, a new technique called MAP was developed^8^. MAP enables super-resolution imaging of intact-tissue multiscale organization with a tissue-hydrogel hybrid^8^. The MAP method linearly expands entire organs four-fold while, like ExM preserving their overall architecture and three-dimensional proteome organization^7,8,11,12^. The key process of the MAP technique is high temperature denaturation and dissociation, which is an effective and unique way to expand tissues. However, high temperature treatment leads to limitations in the imaging of fluorescent-tagged proteins^8^.

In this study, we introduce a new technique we call “Cell-MAP”, which enables multiscale super-resolution imaging of such elements as subcellular architectures and allows for ultrastructural characterization, and for determining the molecular identity of single cells. Optimized-Cell-MAP is a modified and optimized Cell-MAP technique for cell-specific super-resolution imaging that preserves fluorescence. Optimized-Cell-MAP performs stable super-resolution imaging analysis through an intact cell-hydrogel hybrid that induces the expansion of the complete cellular composition and subcellular structures approximately four to five-fold, like MAP, but without high-temperature denaturation and destruction of fluorescent-tagged proteins.

## Results

### Development of a cellular expansion and transparency technique based on MAP

**Figure 1:**
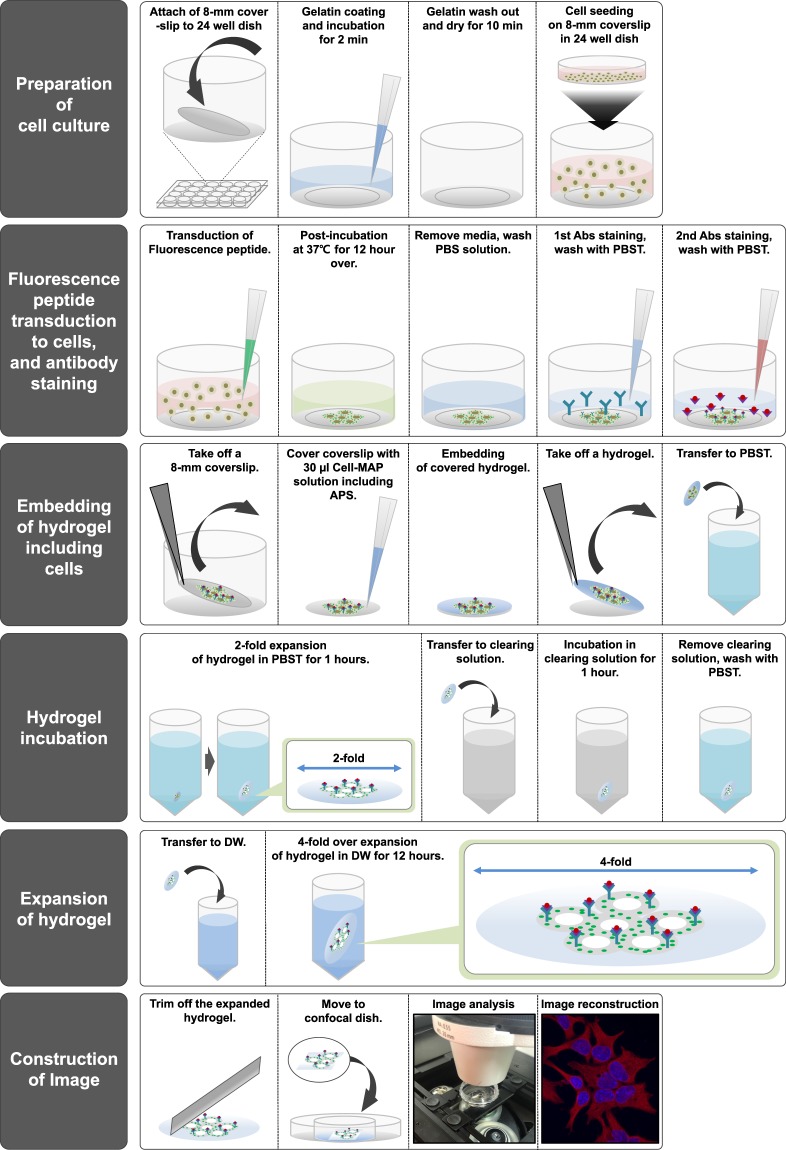
Schematic representation of Cell-MAP processing using fluorescence peptides. The Cell-MAP procedure, including staining and imaging: (1) Preparation of cell culture, (2) Fluorescence peptide transduction to cells, (3) Embedding of hydrogel, including cells, (4) Hydrogel incubation and staining, (5) Expansion of hydrogel, (6) Construction of imaging.

**Figure 2:**
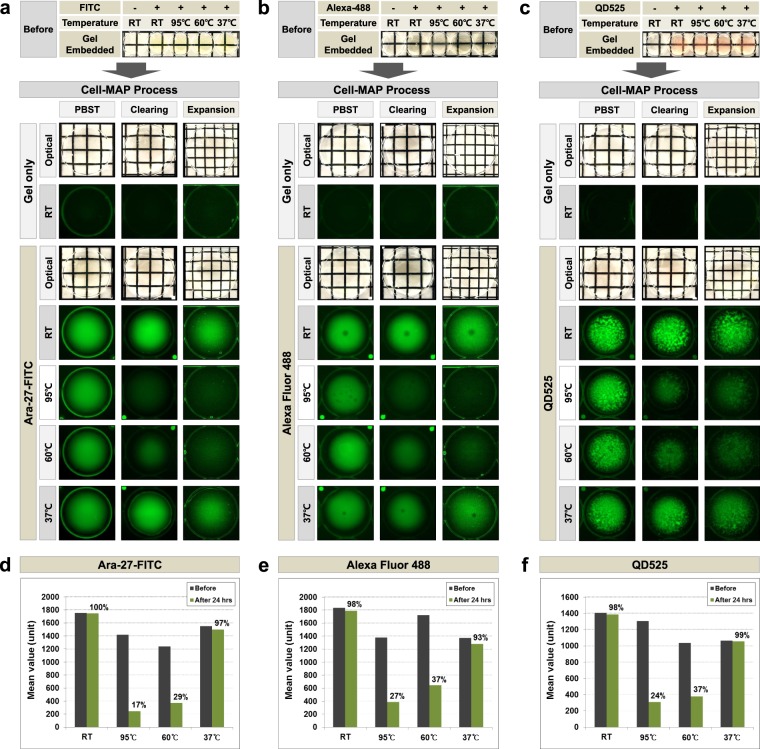
Comparison of fluorescence stability as influenced by temperature, using Cell-MAP gels. A comparison of fluorescence preservation using hybrid gels including the (**a**) Ara-27-FITC, (**b**) Alexa Fluor 488 dye and (**c**) QD525 with incubation of different temperatures (RT, 95 °C, 60 °C and 37 °C) by the whole Cell-MAP process. A comparison of the analyzed fluorescence preservation efficiency of (**d**) FITC (Ara-27-FITC), (**e**) Alexa Fluor 488 dye and (**f**) QD525 hybrid gels on fluorescence images of (**a**–**c**) at after 24 hours of incubation. The transparency of the transparent Cell-MAP gels was evident against a patterned background (length:width = 5 mm:5 mm).

**Figure 3:**
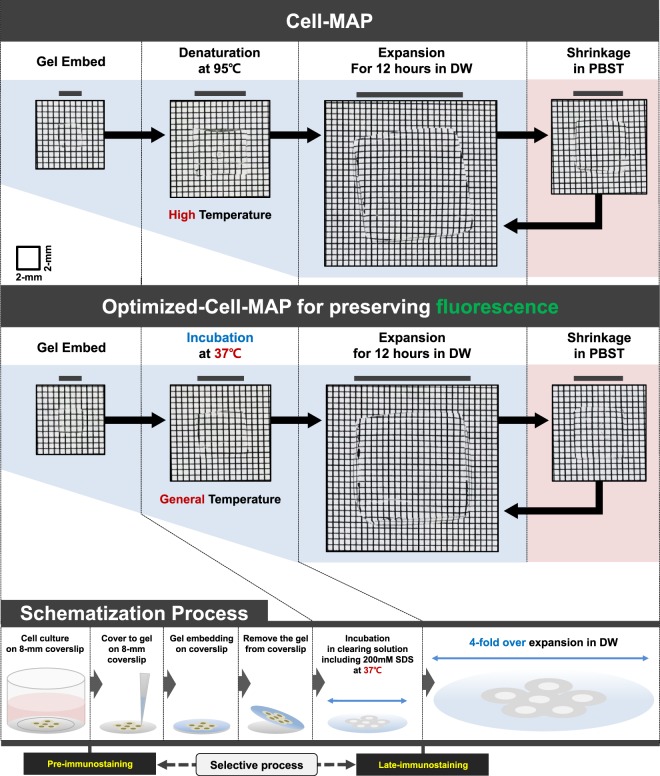
Schematic depictions of the process steps of the Cell-MAP methods. A comparison of hydrogel-cell hybridization and hybrid expansion between Cell-MAP method (up column) and the Optimized-Cell-MAP method for preserving fluorescence (down column). The transparency of all cleared or expanded samples was visualized against a patterned background (length:width = 2 mm:2 mm) to improve contrast.

In this work, we introduce Cell-MAP for cell super-resolution imaging that preserves fluorescence allows the cell specimen to expend more than four-fold via hydrogelation **(**Fig. 1 and Supplementary Fig. 1**)**. In the original MAP, the analysis of samples pre-treated with peptide or antibodies with a fluorescent substance was hindered by loss of fluorescence, which was an obstacle to data analysis. To address this technical problem, we hypothesized that the denaturation step of samples at high temperature (i.e., 95 °C) in the original MAP and Cell-MAP protocols caused fluorescence loss. To verify our hypothesis, we evaluated the loss of fluorescence at high temperature. As shown in Fig. 2, we generated various spherical hydrogels based on gel compositions of a mixture (Cell-MAP solution) of 20% AA, 0.1% bis-acrylamide, 10% sodium acrylate (SA) and 0.65% TEMED with three fluorescence substances, including FITC (Fluorescein isothiocyanate; Ara-27-FITC) peptide, Alexa Fluor 488 dye and QD525 (Quantum Dot 525). The fluorescence stability of three fluorescent MAP gels was compared by incubating at four different temperatures (room temperature [RT], 37 °C, 60 °C and 95 °C) for 24 hours in clearing solution, and observed the fluorescence preservation after the whole Cell-MAP process. In the result, after 24 hours, the fluorescence of the Cell-MAP gels greatly differed among the four temperatures. The samples incubated at RT and 37 °C lost <10% of their fluorescence whereas 60–80% of fluorescence was lost in the samples incubated at 60 °C and 95 °C **(**Fig. 2d–f**)**. These results confirm that the 95 °C denaturation step in the original MAP and Cell-MAP process is related to fluorescence loss. In the newly developed Cell-MAP procedure (Optimized-Cell-MAP), incubation and clearing was performed at 37 °C was performed rather than at the higher temperature of the standard procedure. Both Cell-MAP and Optimized-Cell-MAP for preserving fluorescence methods show gel expansion of four-fold or more in the same type of sample **(**Fig. 3**)**. Together this shows that the Cell-MAP approach clarifies and enlarges cells four fold or more at lower temperatures and retains cell fluorescence.

**Figure 4:**
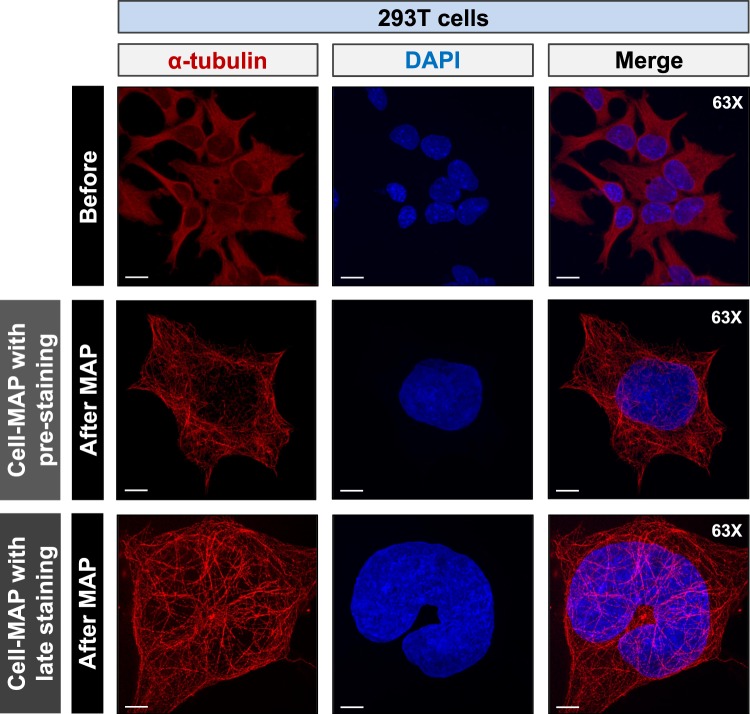
Comparison of multiscale architectures before and after Cell-MAP processing. 293T cells stained for alpha-tubulin and imaged before and after Cell-MAP processing. Cells that stained with alpha-tubulin (red) indicate a cytoskeleton. DAPI (blue) was used to label nucleic acids. Each image (before and after Cell-MAP) was taken with the same 63x object lens and z-stacked for comparison. Scale bars, 15 μm (white).

We next compared and visualized 293T cells before and after Optimized-Cell-MAP processing. The expanded cells were stained with the nuclear marker DAPI (4′,6-diamidino-2-phenylindole) dye and the cell cytoskeleton marker α-tubulin. Imaging analysis was then performed using a 63x objective on a confocal laser microscope. After Optimized-Cell-MAP was performed, the sample measurement sensitivity at the same magnification of 63x was observed to be four times greater than that of the single cell state **(**Fig. 4 and Supplementary Fig. 2**)**. The nucleus and cytoskeleton were clearly observed in clear and super-resolution images after applying Optimized-Cell-MAP **(**Supplementary Video 1**)**. Morphological structures and molecular units were observed at higher resolution compared with conventional confocal imaging. Image analysis performed at 63x and 40x magnification revealed that the image sharpness of the Golgi (stained for giantin) and lysosomes (stained for LAMP-1: lysosomal-associated membrane glycoprotein) was improved and that the images were enlarged more than four-fold. This increase in magnification allowed for clear observation of cellular structures **(**Supplementary Fig. 3**)**. These results demonstrate that our Optimized-Cell-MAP technology is an effective and easy-to-use research tool for the super-resolution imaging analysis of intracellular structural features.

### Image analysis for the efficiency of peptide transfection using Cell-MAP

**Figure 5:**
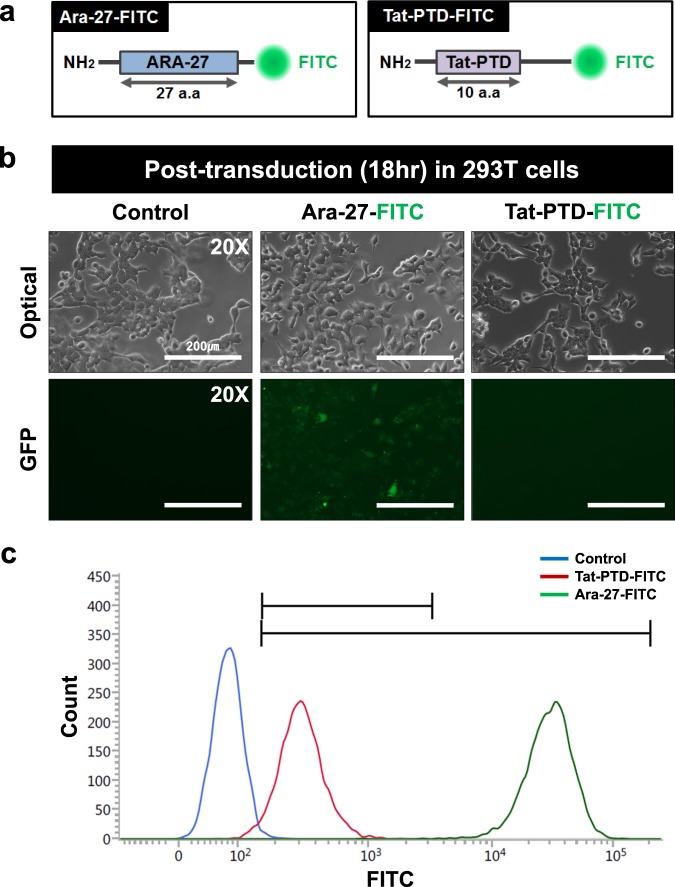
Results of 293T cell fluorescence microscopy and FACS analysis. (**a**) Schematic diagrams of Ara-27 based synthesized peptides, including Ara-27-FITC and Ara-27-ISP-FITC. Ara-27 (blue) consists of a 27 amino acid sequence and was fused with FITC. ISP (pink) is composed a peptide of 24 amino acid, and was fuse inserted into the center of Ara-27-FITC. (**b**) Comparison of transduction efficiency of 18 hour post-transducted 293T cells with Ara-27-FITC and Tat-PTD-FITC peptides. All figures were captured with low magnification (20x) air lenses. Scale bars, 200 μm (white). (**c**) Analyzed transduction efficiency of (**b**) by FACS. Each line and pick were specific to only 293T cells (blue), transducted 293T cells with Tat-PTD-FITC peptide (red) and transducted 293T cells with Ara-27-FITC peptide (green).

To further validate Optimized-Cell-MAP, we analyzed images of cells transfected with heterogeneous foreign materials. We developed the Ara-27 peptide for use as a cell membrane penetrating peptide for a molecule delivery method **(**Supplementary Table 1**)**. This tool involves the passing of a heterologous substance from outside a cell through the cell membrane and into the cytosol. We analyzed the cell membrane penetrating efficiency of peptide transfection into 293T cells using a FITC/fluorescence conjugated Ara-27 (Ara-27-FITC) peptide. The cells were transfected with Ara-27-FITC and Tat-PTD-FITC for 18 hours and we compared the degree of transfection of peptides in the cells. Fluorescence microscopy imaging revealed that the Tat-PTD-FITC treated group was barely transfected and did not show FITC in the cells, whereas the Ara-27-FITC treated group showed strong FITC transfection **(**Fig. 5 and Supplementary Fig. 4a**)**. The intracellular delivery efficiency of the Ara-27 in primary cultured dorsal root ganglia (DRG) cells was similar to the results observed in 293T cells **(**Supplementary Fig. 4b**)**. Fluorescence-activated cell sorting (FACS) analysis of the transfection efficiency of the two peptides in 293T cells showed that the transfection efficiency of the Ara-27-FITC was much higher than that of the Tat-PTD-FITC **(**Fig. 5c**)**. Together, this shows that the new peptide material has a much higher intracellular permeability than conventional Tat-PTD peptides.

In addition, we evaluated the fluorescence preservation efficiency of the Ara-27-FITC peptide in 293T cells through incubation in four different temperatures conditions (RT, 37 °C, 60 °C and 95 °C) in whole Cell-MAP process **(**Supplementary Fig. 5**)**. In the result, the Ara-27-FITC demonstrated high fluorescence preservation efficiency in hybrid 293T cells, similar to the previous results (cf. Fig. 2a,d**)** of the 37 °C incubation in the Cell-MAP gel process for preserving fluorescence. These results suggest that the Optimized-Cell-MAP the improves stability of fluorescent substances in cells through the 37 °C incubation of hybrid-gel, enabling three-dimensional imaging of the super-resolution in cells using a confocal laser microscope.

**Figure 6:**
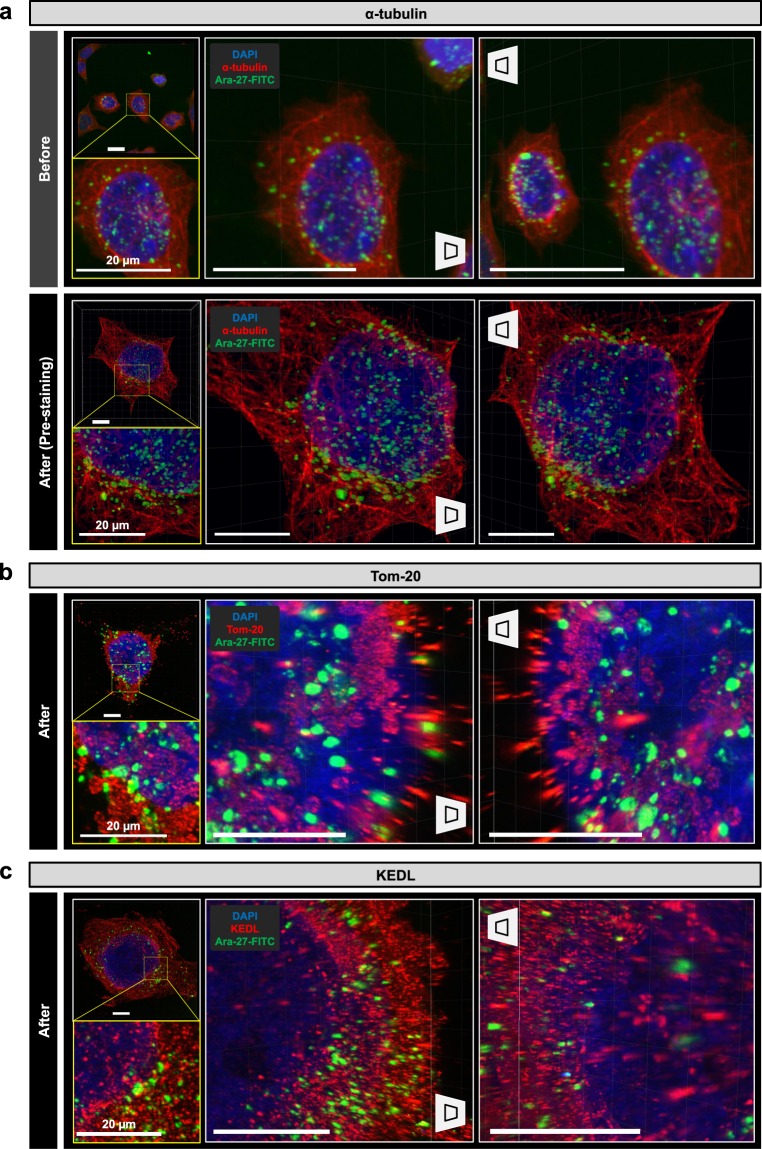
Constructed three-dimensional images of transducted 293T cells with Ara-27-FITC peptide. (**a,b**) Ara-27-FITC treated 293T cells stained for alpha-tubulin and imaged before and after Cell-MAP processing. (**c,d**) Three-dimensional rendering of Tom 20 and KEDL (endoplasmic reticulum marker) images of after Cell-MAP in Ara-27-ISP-FITC peptide treated 293T cells. DAPI was used to label nucleic acids. DAPI (blue), a-tubulin (red), Tom-20 (mitochondria marker) (red), KEDL (red) and Ara-27-FITC (green). Scale bars, 20 μm (white).

The distribution of the Ara-27-FITC in the intracellular location was reconstructed and analyzed by super-resolution three-dimensional imaging. The cell cytoskeleton (stained for α-tubulin) images and the intracellular Ara-27-FITC image produced via Optimized-Cell-MAP processing as well as the picture produced by our protocol were much clearer than conventional confocal microscopy at 63x **(**Fig. 6a and Supplementary Video 2**)**. The Optimized-Cell-MAP imaging evaluation of the U87MG Glioblastoma cell line showed results similar to those of the cell cytoskeleton study **(**Supplementary Fig. 6**)**. We obtained a super-resolution image of mitochondria magnified over four times using a Tom20 (mitochondria marker) antibody. The intracellular location of Ara-27-FITC was then analyzed through a validated image. There was no overlap between Ara-27-FITC and the mitochondria **(**Fig. 6b and Supplementary Video 3**)**. An endoplasmic reticulum super-resolution image was then obtained by a KDEL (endoplasmic reticulum marker) antibody staining. No significant overlap of the Ara-27-FITC transfer with the endoplasmic reticulum could be identified **(**Fig. 6c**)**. These results confirm that the transfection of the Ara-27-FITC effectively permeated the cell membrane but not the intracellular organelles or nuclei. Thus, our new technique has two important features: (i) Optimized-Cell-MAP enable super-resolution imaging analysis of intracellular organelles and peptides; (ii) Optimized-Cell-MAP can maintain the fluorescence of a fluorescent substance at a stable level.

### Analysis of the intracellular location of regenerated peptides using Cell-MAP

**Figure 7:**
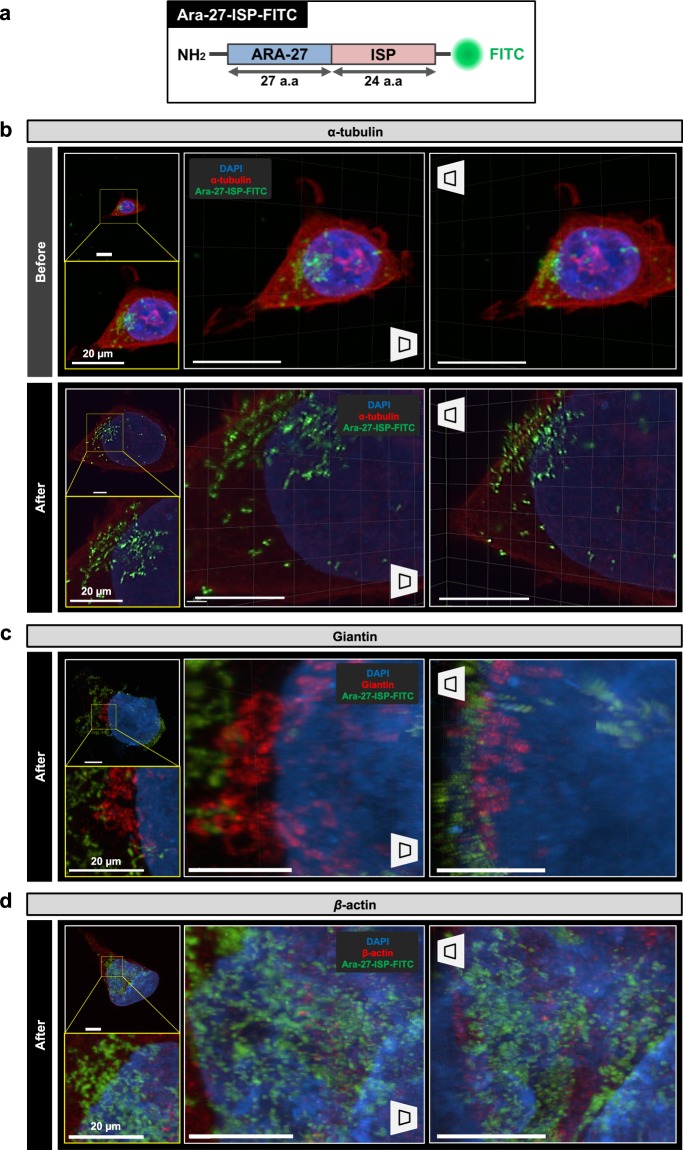
Constructed three-dimensional images of transducted 293T cells with Ara-27-ISP-FITC peptide. (**a**) Comparison of multiscale architectures before and after Cell-MAP processing in Ara-27-ISP-FITC treated 293T cells. Ara-27-ISP-FITC treated 293T cells stained for alpha-tubulin and imaged before and after Cell-MAP processing. (**b,c**) Three-dimensional rendering of tubulin images before and after in Ara-27-ISP-FITC peptide treated 293T cells. (**d,e**) Three-dimensional rendering of giantin and *β*-actin images after Cell-MAP in Ara-27-ISP-FITC peptide treated 293T cells. DAPI was used to label nucleic acids. DAPI (blue), alpha-tubulin (red), giantin (golgi marker) (red), *β*-actin (actin marker) (red) and Ara-27-ISP-FITC (green). Scale bars, 20 μm (white).

Analysis of functional changes within the cell was conducted by attaching the Ara-27-FITC peptide to a new synthetic peptide with a specific sequence. The new peptide, Ara-27-ISP-FITC was synthesized by conjugation of the Ara-27 peptide with a 24-amino acid sequence of intracellular sigma peptide (ISP), a peptide-mimetic of PTP δ (protein tyrosine phosphatase) **(**Supplementary Table 1 and Fig. 7a**)**^13^. We analyzed the intracellular influx of the Ara-27-ISP-FITC by transfecting 293T cells with peptide and performing Optimized-Cell-MAP. Image analysis was performed after α-tubulin staining. Unlike the slightly dimmed image at 63x magnification before Optimized-Cell-MAP, clear and sharp super-resolution images of intracellularly delivered peptides were obtained with Optimized-Cell-MAP. However, there was no interaction between the cytoskeleton and the Ara-27-FITC peptide **(**Figs. 6a and 7b**)**. Giantin (a Golgi marker) staining was performed to analyze the interaction between Golgi bodies and the Ara-27-ISP-FITC peptide. Ara-27-ISP-FITC was located independent of the Golgi, indicating that Golgi are not associated with Ara-27-ISP-FITC movement and function **(**Fig. 7c and Supplementary Video 4**)**. In addition, the relationship between *β*-actin (actin marker) and cell permeability was analyzed and showed similar results to those associated as with Golgi (Fig. 7d**)**. Ara-27-ISP-FITC was located separately from the *β*-actin. These image analysis results indicate that the Ara-27 and Ara-27-ISP peptides did not interact with any organelles in the cell during delivery into the cell. The Ara-27 peptide is presumably an independent molecule that permeates the cell membrane, enters the cell, and operates independently. These results demonstrate the technical capabilities of Optimized-Cell-MAP, which can easily make subcellular organelles visible by increasing their size four-fold or more and maintaining the characteristics of pre-tagged fluorescent proteins.

### Application of Cell-MAP for the imaging of micro-RNA

**Figure 8:**
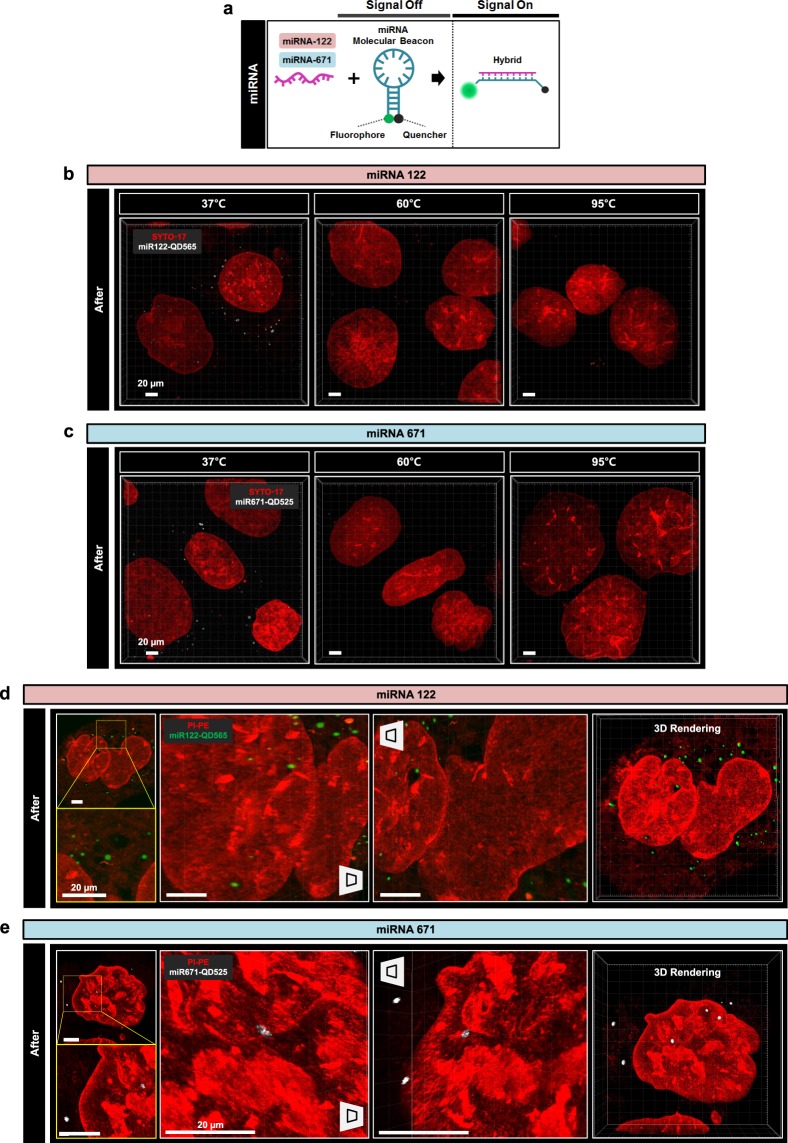
Comparison of multiscale architectures before and after Optimized-Cell-MAP processing in micro-RNA treated Hep3B cells. (**a**) A schematic diagram of the molecular beacon (MB) assay. The complementary region for the target miRNA is located between the 3′-end quencher and the stem loop region for signal on/off. A schematic illustration of miRNA monitoring using an MB is shown. The mature miRNAs are marked in purple, and the MB probes are marked in blue green. Fluorescence signals of the MB probe against different target miRNAs, including miR-122 and miR-671 (purple). (**b,c**) A comparison of QD fluorescence preservation using MB probes of miR-122 and miR-671 with incubation at different temperatures (RT, 37 °C, 60 °C, and 95 °C) by the optimized-Cell-MAP process. 3D projection images of SYTO-17 (red), miR122-QD565 (white), and miR671-QD525 (white). (**d**) miR-122 treated Hep3B cells stained with PI-PE and imaged after Cell-MAP processing. 3D projection images of PI-PE (red) and miR122-QD565 (green). (**e**) MiR-671 treated Hep3B cells stained with PI-PE and imaged after Cell-MAP processing. 3D projection images of PI-PE (red) and miR671-QD525 (white). Scale bars: 20 μm (white).

Using Optimized-Cell-MAP for preserving fluorescence, we analyzed FITC-tagged peptides in transfected cells and confirmed the stability of fluorescent material such as FITC. We next attempted to verify that our Cell-MAP procedure is stable against other fluorescent materials as quantum dot and inside cells by super-resolution imaging analysis. Specifically, we investigated whether subcellular components (e.g., microRNA), which are not well detectable by conventional confocal microscopes, could be observed using our technique. We treated cells with an molecular beacon (MB) probe that can detect fluorescence only when hybridized to a target, and then analyzed the intracellular pattern through Optimized-Cell-MAP. We used microRNA-122 (miR-122) and microRNA-671 (miR-671), which are known to induce apoptosis in hepatoma cells **(**Fig. 8a**)**^14–19^. Two types of miRNA-specific MBs were used to analyze the expression patterns in hepatocellular carcinoma cells (Hep3B cell line) after Cell-MAP imaging. miR-122- and miR-671-linked MB containing QD565 and QD525 were transfected into Hep3B cell line. The levels of the two miRNAs in Hep3B cells stained with SYTO-17 dyes and propidium-iodide phycoerythrin (PI-PE) were analyzed with Optimized-Cell-MAP.

miR-122, as shown by the binding reaction with MB, was observed to be dispersed into several dot images in the cytoplasm by super-resolution imaging analysis **(**Fig. 8b,d**)**. Likewise, miR-671 was detected in the cytoplasm. However, miR-671 was less pronounced than miR-122 **(**Fig. 8c,e**)**. In addition, the level of two miRNAs in Hep3B cells was shown to express stably, and fluorescence of QDs was preserved by the Optimize-Cell-MAP process of 37 °C incubation compared to high temperatures (60 °C and 95 °C) **(**Fig. 8b,c**)**. These results, found through Optimized-Cell-MAP for preserving fluorescence, indicate that miR-122 and miR-671 are localized in the cytoplasm of hepatocellular carcinoma cells. In addition, this reveals that Optimized-Cell-MAP is a viable imaging technology for quantum dot signals in cells for microRNAs imaging.

## Discussion

The MAP technique allows more than four-fold expansion (while preserving the clarity) of a hydrogel hybrid, which enables super-resolution imaging and analysis of the overall architecture and three-dimensional proteome of a multiscale organization^8^. Here we introduce Cell-MAP, which is a modified, cell-specific MAP that preserves fluorescence stability. The Cell-MAP method expands hydrogel-embedded cells four-fold while preserving their overall architecture and three-dimensional proteome organization. In addition, Optimized-Cell-MAP has the ability to preserve fluorescence, allowing the observation of tagged small molecular probes containing peptides and microRNAs.

To overcome fluorescence probe disappearance at high temperatures, protocol optimization was performed to preserve fluorescent probes (i.e., FITC, Alexa Fluor 488 and Quantum dot 525) in the cell after the MAP process. Cell-MAP employs low temperature (37 °C) clearing rather than the high temperature (60 °C and 95 °C) used in the original MAP protocol. The denaturation and dissociation of tissue at high-temperatures after preventing intra- and interprotein crosslinking with high concentrations of acrylamide is the key step of MAP^8^. PFA (paraformaldehyde) generates intra- and interprotein crosslinks that makes tissues stable, but a strong crosslink can prevent tissue expansion. MAP uses high concentrations (20–30%) of acrylamide to prevent crosslinking and denaturation with high temperature for effective expansion. In this study, we showed the different fluorescence preservation efficiencies at four different temperatures (RT, 95 °C, 60 °C and 37 °C) in the whole Cell-MAP process with three fluorescence substances, including FITC peptide, Alexa Fluor 488 and QD525.

Optimized-Cell-MAP was developed for super-resolution imaging analysis by fluorescence preservation via cell-specific clearing and expansion. This method is applicable at levels ranging from a single cell to that cell complex, such as an organoid. However, when using a complex tissue, the low temperature (37 °C) application of the Optimized-Cell-MAP process may be incompatible. For tissue and organ expansion, denaturation is essential, but this step is not required for cell samples. Optimized Cell-MAP does not have a dissociation step and instead just incubates and removes lipids via clearing solution application at a low temperature (37 °C). Clearing alone is sufficient. Thus, we have developed a simple and scalable cell super-resolution imaging assay for subcellular structure 3D proteome imaging.

Using Optimized-Cell-MAP for preserving fluorescence, we observed the nucleus, cytoskeleton, various organelles and miRNA of a cell through super-resolution imaging that clearly showed cell structures and reduced the level of background noise in all images. Cell-MAP produced super-resolution images but at a lower resolution than super-resolution microscopies such as PALM, STORM, and STED, which cannot image live cells^20–22^. Despite these limitations, Cell-MAP has several advantages over super-resolution microscopy. The entire Cell-MAP process, including immunostaining and imaging, can be accomplished in one day, and does not require a special and expensive super-resolution microscope. Only a conventional confocal microscope is required, and all kinds of conventional antibodies can be utilized with Cell-MAP. In addition, compared with ExM^7,11^ and ultrastructure expansion microscopy (U-ExM)^12^, sample preparation for Cell-MAP is much simpler. ExM also requires 60 °C incubation of samples, which will inevitably eliminate cell flourescence.

Using super-resolution imaging by Optimized-Cell-MAP, we demonstrated the ability to localize cell membrane penetrating peptides and miRNA. Small peptide and miRNA size is limited to observation with a conventional confocal microscopy. We observed the new cell membrane penetrating Ara-27-FITC and Ara-27-ISP-FITC peptides transfering into the cytosol after penetrating into the cell and interact with the cytoplasm but not with the nucleus, cytoskeleton, mitochondria, endoplasmic reticulum (ER), Golgi or actin. We hypothesize that these Ara-27 peptides infiltrate into the cytoplasm independently, without any effect on cell organelles, and achieve functional activation mainly within the cytoplasm.

Finally, we analyzed the expression of miR-122 and miR-671 in hepatocellular carcinoma cells after treatment with a specific MB^16,17,23,24^. We successfully observed the intracellular distribution and expression patterns of microRNAs in hepatoma cells, which are similar to those previously reported^25^. However, the resolution in the present study was higher than in any previous study^25^. In addition, we detected miR-671 in hepatocellular cells and miR-671 that had not previously been reported. The ExFISH technique, a combination of ExM and FISH, has been reported for RNA imaging^26^. ExFISH is more complicated and will not preserve fluorescence because of the 60 °C incubation. The QD is known as having permanence by guarantee constant fluorescence in any environment. In contrast, in the Cell-MAP process at four different temperatures, QD was shown to lose fluorescence after incubation at high temperature (60 °C and 90 °C) but to be stable at a low temperature. However, this may represent probe loss in the Cell-MAP gel due to high temperature condition although the probes have not lost their true light intensity. Thus, Optimized-Cell-MAP using MB is a very simple and stable miRNA imaging tool. Optimized-Cell-MAP is shown to be a breakthrough cell enlargement transparency technique that can be used to observe the structural characteristics of cells and the functionality of substances delivered into cells. As a new imaging analysis technology, Optimized-Cell-MAP goes beyond the limits of current confocal imaging analysis.

## Materials and Methods

### Animals

Adult male Sprague Dawley (SD) rats (200–250 g, 4-week-old) were purchased from Koatech Inc. (Gyeonggi-Do, Korea). This study was carried out in strict accordance with the recommendations in the Guide for the Care and Use of Laboratory Animals of the Ministry of Agriculture, Food and Rural Affairs (MAFRA) and approved by the Institutional Animal Care and Use Committee (IACUC) of the University of Yonsei University (#2017-0230 and #2015-0147). All animal procedures were conducted under veterinarian supervision according to the guidelines imposed by the Ethical Committee. After anesthesia with 2% isoflurane, the thorax of rat was opened, and an incision was made in the right atrium of the heart. Perfusion washing was performed with 250 ml of cold 0.1 M PBS using a 50 mL syringe. The DRG of SD-rat was harvested following standard methods^27,28^, and primary DRG cells of SD-rat were harvested following standard methods^29,30^.

### Cell culture

DRG cells, 293T and Hep3B cell lines were obtained from the American Type Culture Collection (Manassas, VA, USA) and cultured in basic DMEM or DMEM/F12 (Gibco Inc., CA, USA). They were supplemented with 10% fetal bovine serum (Gibco Inc., CA, USA) and incubated under a 5% CO_2_ atmosphere at 37 °C. Round glass coverslips (8-mm) were coated with 0.1% gelatin in ultrapure water (Millipore Inc., MA, USA) and placed in a 24-well plate; the wells were seeded with 1.5 × 10^4^ cells overnight.

### Synthesis and preparation of cell membrane penetrating peptide

Various cell membrane penetrating peptides were synthesized by LifeTein LLC (NJ, USA). Tat-PTD was made by a previously reported method^31^. We developed the new cell membrane penetrating peptide “Ara-27” for molecule delivery method. Ara-27-ISP was synthesized by conjugation of Ara-27 with a 24-amino acid sequence of intracellular sigma peptide (ISP), a peptide-mimetic of PTP δ (protein tyrosine phosphatase)^13^. Tat-PTD, Ara-27, and Ara-27-ISP were conjugated with fluorescein isothiocyanate (FITC) using lysine (K) at the C-terminus. The sequences of the peptides used in this study are summarized in Supplementary Table 1. Tat-PTD-FITC, Ara-27-FITC, and Ara-27-ISP-FITC were dissolved in DW and then diluted to the desired concentrations.

### microRNA molecular beacon (MB) conjugated with nanoparticle probes

To prepare the molecular beacon (MB) for the *in vitro* experiment, we designed a single-stranded oligonucleotide with amine parts at the cohesive end, and then linked it with black hole quencher (BHQ) at the 3′-end^23,24,32^. QD525-COOH and QD565-COOH nanoparticles were purchased from Molecular probe (ThermoFisher, Waltham, USA)^23,24,32^. To construct QD-miR-122 MB and QD-miR-671 MB, two oligonucleotides were synthesized by Bioneer Inc (Daejeon, Korea). The miRNA-122 MB and miR-671 MB were formed as partly double stranded oligonucleotides following a previous report^24,32^. The miR-671-linked MB contains QD525 (excitation/emission wavelength: 460/525 nm) and BHQ-2. The designed miR-122-linked MB contains QD565 (excitation/emission wavelength: 565/625 nm) and BHQ-1. The sequences of miRNA MBs used in this study are summarized in Supplementary Table 2. The MBs with sequences complementary to mature miR-122 or miR-671 were designed and synthesized^33^.

### Transfection of peptides and fluorescence microscopy imaging

DRG and 293T cells of 1 × 10^5^ cells were suspended in 4 ml of DMEM or DMEM/F12 media (Gibco Inc., CA, USA) and seeded in 6-well plates. Next, 2.5 µl of 5 mM Ara-27-FITC peptide was added to each well. After incubation at 37 °C for 18 hours, the media was removed and replaced with fresh media. FITC-positive DRG and 293T cells were then observed by fluorescence microscopy (EVOS® FL Cell Imaging System, Invitrogen Inc., CA, USA).

### FACS analysis

293T cells were seeded in 6-well plates at a density of 6.0 × 10^5^ cells per well. After 24 h, the fluorescence peptides were treated to the culture medium (5 μM of Tat-PTD-FITC, 5 μM of Ara-27-FITC) and incubated at 37 °C for 90 min. The cells were washed three times with PBS containing heparin (Sigma-Aldrich Inc., MO, USA) and harvested using 0.05% trypsin. Isolated single cells were washed and resuspended in PBS containing 5% BSA. The cells were analyzed by FACSVerse (Becton Dickinson, Franklin Lakes, NJ, USA).

### Immunocytochemistry analysis

To obtain comparable images before and after Cell-MAP processing, cells were washed, fixed with 4% PFA in PBS for 10 min, and switched to a solution of 4% PFA and 20% acrylamide in PBS for 8 h at 37 °C. Cells were then placed in 0.1% sodium borohydride for 7 min at RT and incubated in 100 mM glycine for 10 min at room temperature (RT). Cells were washed and sequentially stained with primary antibodies, secondary antibodies, and DAPI (Invitrogen Inc., CA, USA). Finally, cells were mounted in 2,2′-thiodiethanol (Sigma-Aldrich Inc., MO, USA) and imaged with a 63x, 1.3 NA glycerol-immersion objective with an LSM780 confocal laser scanning microscope (Cal Zeiss, Jena, Germany) using 10x, 20x, 40x and 63x magnifications and internal Zeiss software.

### MAP technique

#### Original MAP and Cell-MAP

Cells were thoroughly washed and embedded into a hybrid polymer by adding 30 μL of MAP solution (20% acrylamide (AA), 7% sodium acrylate (SA), 0.1% bis-acrylamide (BA), 0.5% TEMED, in PBS) or Cell-MAP solution (20% AA, 10% SA, 0.1% BA, 0.65% TEMED, in PBS). Ammonium persulfate (APS) from a freshly prepared 5% stock solution was added to both samples last. The MAP and Cell-MAP solutions were quickly added to the coverslip and left to polymerize for 5 min. The gels were peeled off the coverslip using forceps, washed thoroughly, and incubated for 30 min in clearing solution (200 mM Sodium Dodecyl Sulfate (SDS), 200 mM NaCl and 50 mM Tris in DW) at 95 °C (for Cell-MAP) or incubated for 30 min in clearing solution was executed at 37 °C (for Optimized Cell-MAP). Both original and Cell-MAP gels were incubated until they reached more than 4-fold expansion in DW over 12 hours.

#### Cell-MAP for peptide transfected cells

U87MG and 293T cells (1.5 × 10^4^ cells) were suspended in 0.5 mL of DMEM or DMEM/F12 media (Gibco Inc., CA, USA) and seeded into 24-well plates containing 8-mm round cover slips. Then, 2.5 µL of 5 mM Ara-27-FITC or Ara-27-ISP-FITC peptides were added to the wells. After incubation at 37 °C for 24 hours, cells were fixated with 4% PFA for 15 min. Cells were permeabilized by treatment in 0.2% Triton X-100 (Sigma-Aldrich, Inc., MO, USA) in 0.1 M PBS for 2 hours, and then blocked with a combination of 1% blocking solution of 1% bovine serum albumin (BSA) and 0.3–1 M glycine in 0.1% PBST for 12 hours. Cells were incubated for 1 day with a primary antibody, followed by being washed three times with 0.1 M PBS solution for 2 hours. Next, cells were incubated with a secondary antibody in 1% BSA for 1 day. After washing cells three times with 0.1 M PBS solution for 2 hours, cells were stained with DAPI dye at RT. Cells were washed with 500 µL of PBS and incubated in 300 µL of 4% PFA for 15 min. Next, cells were thoroughly embedded into a MAP hybrid polymer by adding of 30 μL of the Cell-MAP solution. APS (3 µL) from a freshly prepared 5% stock solution was added last. The Cell-MAP solution was quickly added to the coverslip and left to polymerize for 5 min. The gels were then peeled off the coverslip using forceps, washed thoroughly, and incubated for 30 min in clearing solution at 37 °C. The Cell-MAP gels were incubated until they reached 4-fold expansion in DW for 12 hours. The Cell-MAP gels were then cut into pieces, washed thoroughly, and re-stained with the primary antibody and DAPI. A list of all primary and secondary antibodies is shown in Supplementary Table 3.

#### Cell-MAP for MB-treated cells

Hep3B cells (1.5 × 10^4^) were suspended in 0.5 ml of DMEM media (Gibco Inc., CA, USA) and seeded into 24-well plates containing 8-mm round coverslips. Hep3B cells were washed thoroughly and embedded into a hybrid polymer by adding 30 μL of Cell-MAP solution. APS (3 µL) from a freshly prepared 5% stock solution was added last. The Cell-MAP solution was quickly added to the coverslip and left to polymerize for 4–5 min. Gels were peeled off the coverslips using forceps, washed thoroughly, and incubated for 30 min in clearing solution at 37 °C. The gels were incubated with miR-122-QD565 (1:25) and miR-671-QD525 (1:25) in PBST at 4 °C for 2 days. After washing cells three times with 0.1 M PBS solution for 1 hour, the gels were stained with SYTO-17 and propidium iodide phycoerythrin (PI-PE; 1:1000) dyes for 1 hour and DAPI for 5 min. Cells were then incubated for 12 hours until they reached more than 4-fold expansion in DW. The Cell-MAP gels were cut into pieces and washed thoroughly. A list of all primary and secondary antibodies is shown in Supplementary Table 1.

### Fluorescence stability analysis

Hydrogels were embedded into a hybrid polymer with FITC tagged peptides (Ara-27-FITC), Alexa Fluor 488 dye (Alexa Fluor 488 conjugated antibody) and QD525 by adding 30 μL of Cell-MAP solution. APS from a freshly prepared 5% stock solution was added to last both samples last. Two solutions were quickly added to the coverslip and left to polymerize for 5 min. The gels were incubated for 24 hours in clearing solution at different temperatures (RT, 37 °C, 60 °C and 95 °C) for 1 hour in dark conditions. After 24 hours, the hydrogels were transferred to lid of cell culture plates (6-well, 12-well and 96-well) and observed by Gel Documentation System (Gel Doc XR; Biorad Inc., CA, USA).

### Immunostaining and preparation for Cell-MAP imaging

All the clarified or expanded gels that contained cells were incubated with 0.1% Triton X-100 (Sigma-Aldrich, Inc., MO, USA) in 0.1 M PBS for 2 hours and blocked with 2% bovine serum albumin (BSA) in 0.1 M PBS for 6 hours. After washing three times with PBST (0.1% Tween-20 in 0.1 M PBS) solution for 2 hours, immunostaining was carried out for 3 days using a primary antibody. Next, gels were incubated with a secondary antibody in 2% BSA for 3 days. The labeled gel was washed three times with PBST solution for 2 hours and gel expansion was achieved by storing the gel in 30 mL of DW for 1 day in the dark. Before imaging, the labeled gel was moved and fixed with a small amount of DW solution on 35-mm confocal dishes and covered with 20-mm round coverslips. All antibodies are listed in Supplementary Table 1.

### Image analysis

#### Imaging of experiments

Images of Cell-MAP gel placed on each plate (lid of 6-well, 12-well and 96-well cell culture plates) were captured using a digital camera (EOS 100D, Canon Inc., Tokyo, Japan). Ara-27-FITC transfected 293T cells and its hybrid Cell-MAP gels were observed on confocal dish by fluorescence microscopy (EVOS® FL Cell Imaging System, Invitrogen Inc., CA, USA) at 20x magnification.

#### Image processing of 3D reconstruction

Large expanded samples were mounted on confocal dishes. The dishes were filled with dissolved water and the samples were covered with a coverslip. The expanded samples were stabilized for at least one hour before imaging. The samples were acquired by tile scanning using an LSM-780 confocal laser scanning microscope (Cal Zeiss, Jena, Germany) at 40x or 63x magnification and using internal Zeiss software. The three-dimensional images and videos were edited into serial images by Imaris software (Bitplane, Belfast, United Kingdom).

## Supplementary information

Supplementary Information.

Supplementary Video 1.

Supplementary Video 2.

Supplementary Video 3.

Supplementary Video 4.

Check list for submissions.
