# Supplementary material for: A Modified Magnified Analysis of Proteome (MAP) Method for Super-Resolution Cell Imaging that Retains Fluorescence
Source: Sci Rep. 2020 Mar 6;10:4186. doi: 10.1038/s41598-020-61156-2 (PMC7060248; doi:10.1038/s41598-020-61156-2)
Supplement: Supplementary file 1 — Supplementary Information. [file 41598_2020_61156_MOESM1_ESM.docx]

**Supplementary Information**

**Title : A Modified Magnified Analysis of Proteome (MAP) Method for Super-Resolution Cell Imaging that Retains Fluorescence**

**Authors:** Jiwon Woo^1,2*^, Jeong-Min Seo^3,4*^, Mirae Lee^1,2^, Juyoung Kim^1^, Sol Min^3^, Sang-Tae Kim^5^, Seockmo Ku^6^, and Jeong-Yoon Park^1,2¶^

**Affiliations:**

^1^ The Spine and Spinal Cord Institute, Department of Neurosurgery, Gangnam Severance Hospital, Yonsei University College of Medicine, Seoul, 06273, Republic of Korea

^2^ Brain Korea 21 PLUS Project for Medical Science, Yonsei University, Seoul, 03722, Republic of Korea

^3^ Cellular Reprogramming and Embryo Biotechnology Laboratory and Dental Research Institute, Seoul National University School of Dentistry, Seoul, 08826, Republic of Korea

^4^ Biomedical Research Institute, NeoRegen Biotech Co., Ltd., Gyeonggi-do, 16614, Republic of Korea

^5^ Department of Neurology, Seoul National University Bundang Hospital, Seongnam-si, Gyeonggi-do, 13605, Republic of Korea

^6^ Fermentation Science Program, School of Agriculture, College of Basic and Applied Sciences, Middle Tennessee State University, Murfreesboro, TN 37132, USA

**^*^Jiwon Woo & Jeong-Min Seo**. These authors contributed equally to this work

^¶^**Corresponding author:** Jeong-Yoon Park, MD., Ph.D.

Tel: +82-2-2019-3390, Fax: +82-2-3461-9229, E-mail: spinepjy@yuhs.ac

**Supplementary Table 1.** Sequences of cell membrane penetrating peptides.

|  | **Forward** | **Length** |
| --- | --- | --- |
| **Tat-PTD** | GRKKRRQRRK-FITC | 10 |
| **Ara-27** | RNQRKTVRCFRCRQAGHWISDCRLKSK-FITC | 27 |
| **Ara-27-ISP** | RNQRKTVRCFRCRQAGHWISDCRLKSDMAEHMERLK ANDSLKLSQEYESIK-FITC | 51 |
| All peptides were conjugated with fluorescein isothiocyanate (FITC) by adding lysine (K) in the C-terminus. | | |

**Supplementary Table 2.** Sequences of miRNA molecular beacons (MB).

|  | **Sequence** | **Length** | **Probe** |
| --- | --- | --- | --- |
| **miR-122** | 5′-NH^2^-TTCGCTGTCTTTCTCCTTTGTTCTCCTTTCAAGAGACA GATACAGCGCGACAAAGACG-(**BHQ-1**)-3′ | 58 | QD565 |
|  |  |  | -COOH |
| **miR-671** | 5′-NH^2^-TTCGCTGTCTCCTGCCCCTCCTGGGCTTCCTTCAAGAGAC AGATACAGCGGGCAGGAGACAGCG-(**BHQ-2**)-3′ | 67 | QD525 |
|  |  |  | -COOH |
| All miRNA MB were linked with black hole quencher (BHQ-1 and BHQ-2) and carboxylic acid (COOH)-coated quantum dots (QD) at 3'-end of miRNA sequence. | | | |

| Target | Antibody and dye | Vendor | Catalog # | Host species |
| --- | --- | --- | --- | --- |
| Neurofilament | Purified anti-Neurofilament Marker (pan axonal, cocktail) Antibody | Biolegend | 837904 | Mouse |
| Alpha Tubulin | Recombinant Anti-alpha Tubulin antibody, Microtubule Marker | Abcam | ab202272 | Rabbit |
| Giantin | Purified anti-Giantin Antibody | Biolegend | 909701 | Rabbit |
| KDEL | Recombinant Anti-KDEL antibody | Abcam | ab176333 | Mouse |
| Tom20 | Tom20 (F-10) antibody | Santa cruz | sc-17764 | Mouse |
| Actin | Anti-beta Actin antibody | Abcam | ab8227 | Rabbit |
| - | Goat Anti-Rabbit IgG H&L (Alexa Fluor® 488) | Abcam | ab150077 | Rabbit |
| - | Goat Anti-Mouse IgG H&L (Alexa Fluor® 647) | Abcam | ab150115 | Mouse |
| - | Donkey Anti-Rabbit IgG H&L (Alexa Fluor® 647) | Abcam | ab150063 | Rabbit |
| Nuclear | SYTO™ 17 Red Fluorescent Nucleic Acid Stain | Thermo | S7579 | - |
| Nuclear | Propidium iodide-PE | Sigma | P4864 | - |
| Nuclear | DAPI, Hoechst 33342, NucBlue™ Live ReadyProbes™ Reagent | Invitrogen | R37605 | - |

**Supplementary Table 3.** Antibody and dye summary.

| **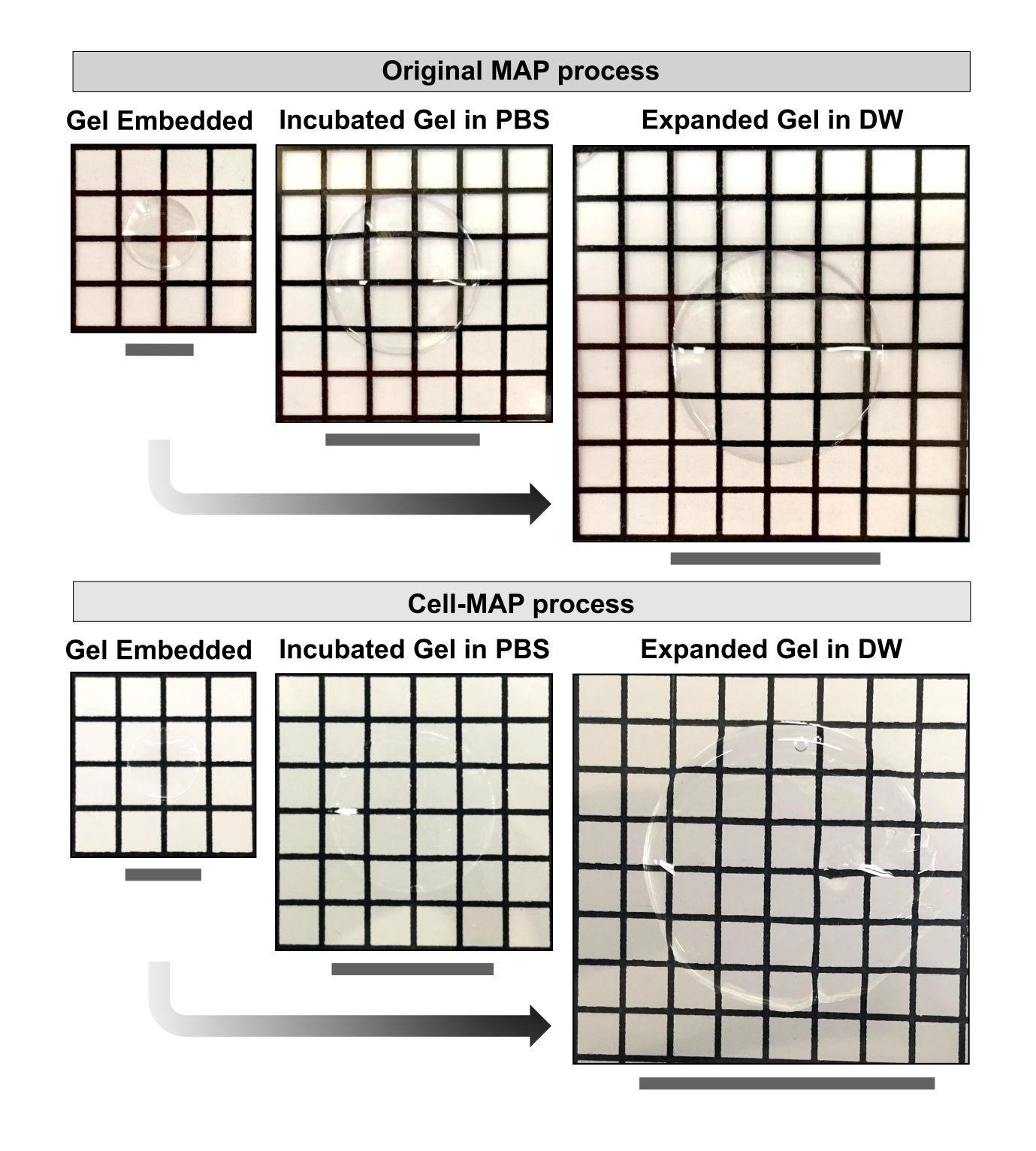** |
| --- |

**Supplementary Figure 1.**

Comparison of hybrid-cells expansion process steps by original MAP and Cell-MAP methods. The transparency of cleared and expanded samples was evident against a patterned background (length: width=5 mm:5 mm). Scale bar (dark grey) indicates gel size.

| **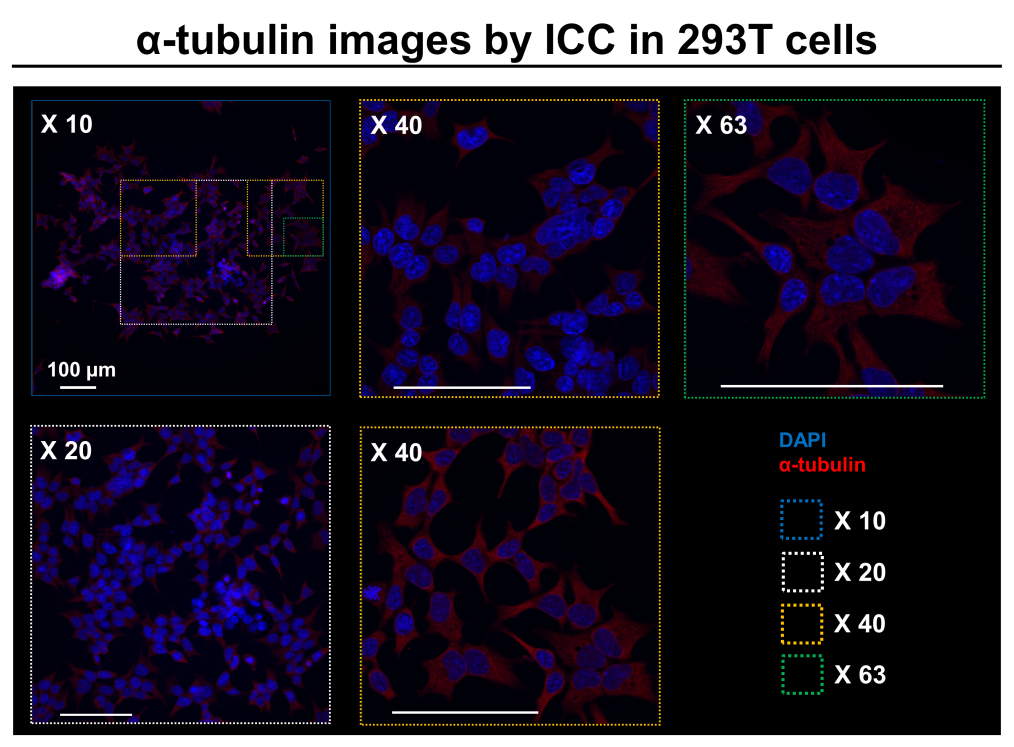** |
| --- |

**Supplementary Figure 2.**

293T cells stained for alpha-tubulin (red) and imaged before Cell-MAP processing. DAPI (blue) was used to label nucleic acids. Squares of dotted lines indicate each different magnification level: 10x (blue), 20x (white), 40x (orange) and 63x (green). Scale bars, 100 μm (white).

| **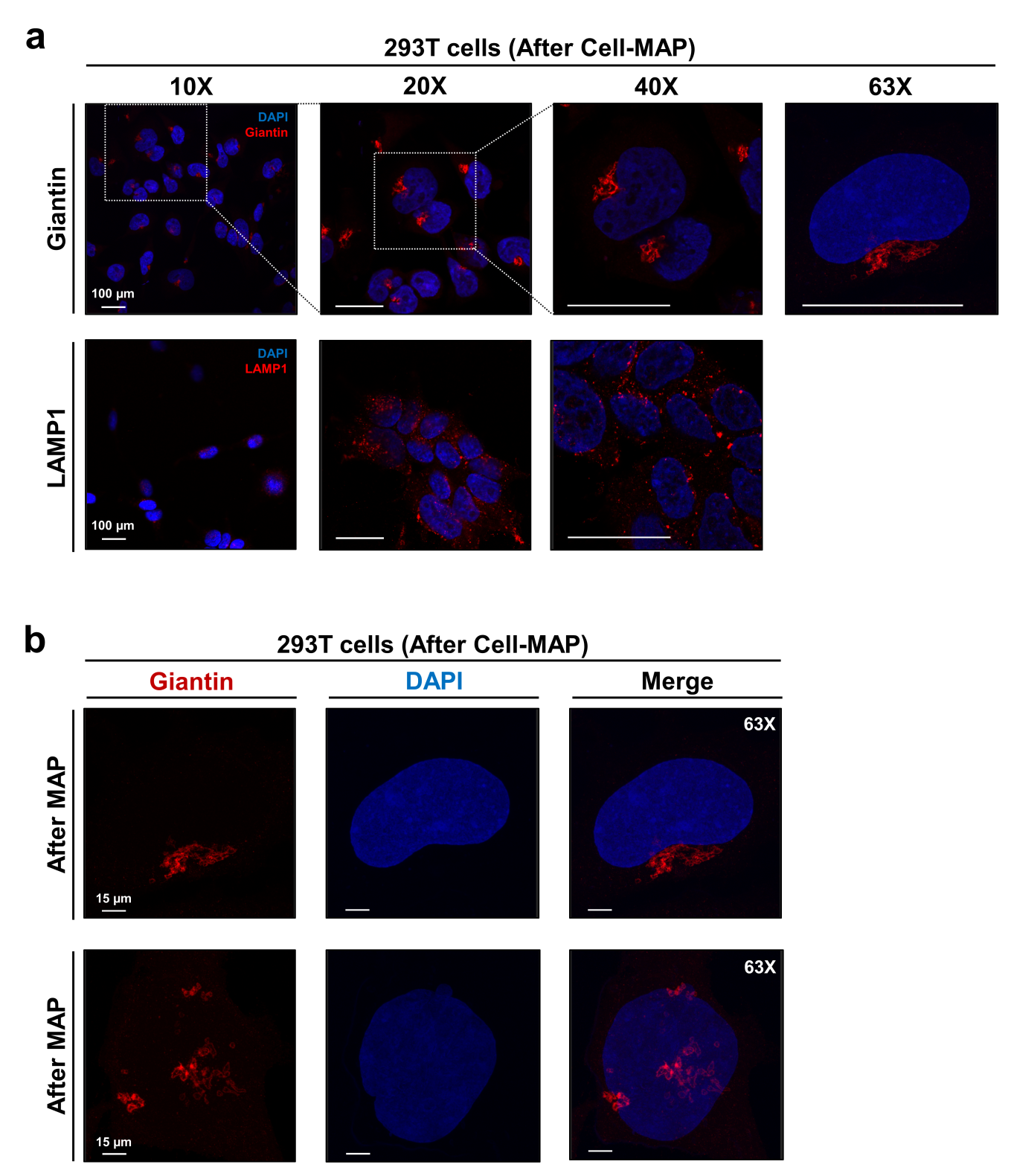** |
| --- |

**Supplementary Figure 3.**

(a) 293T cells stained for giantin (up column) and LAMP1 (Lysosomal-associated membrane glycoprotein 1) (down column), and imaged after Cell-MAP processing. The 293T cells were stained with giantin (red) and LAMP1. Red indicates golgi and lysosome. DAPI (blue) was used to label nucleic acids. Each after Cell-MAP image taken with 10x, 20x, 40x and 63x object lenses was z-stacked for comparison. Scale bars, 100 μm (white). (b) 293T cells stained with giantin (red) and DAPI (blue), and imaged after Cell-MAP processing. Each image was taken after Cell-MAP with same 63x object lens and z-stacked for comparison. Scale bars, 15 μm (white).

| **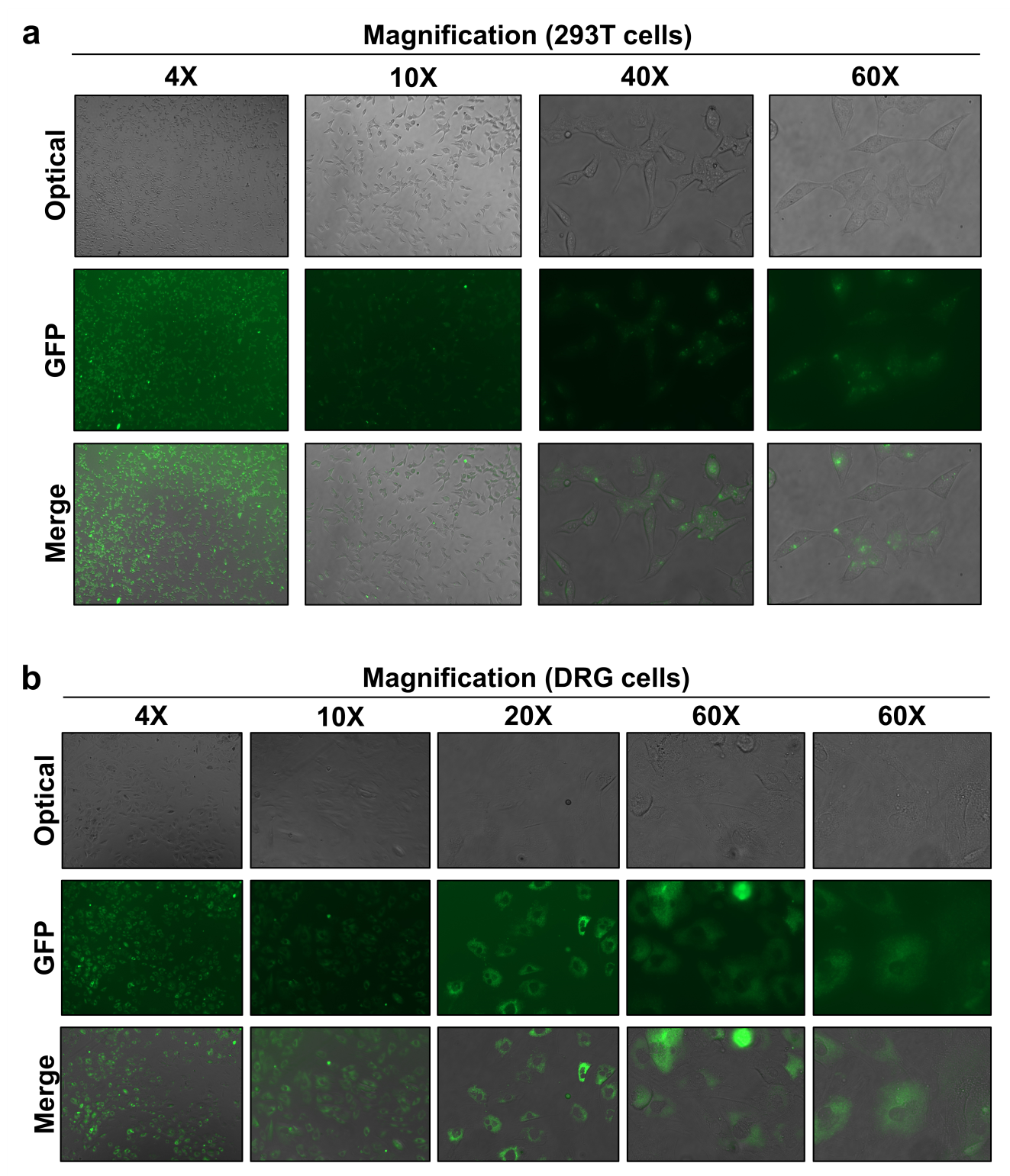** |
| --- |

**Supplementary Figure 4.**

(a) Comparison of transduction efficiency of 18 hour post-transducted 293T cells with Ara-27-FITC peptides (green). (b) Comparison of transduction efficiency of 18 hour post-transducted primary cultured rat DRG cells with Ara-27-FITC peptides (green). All figures were captured with serial magnification (4x, 10x, 20x, 40x and 60x) air lenses.

| **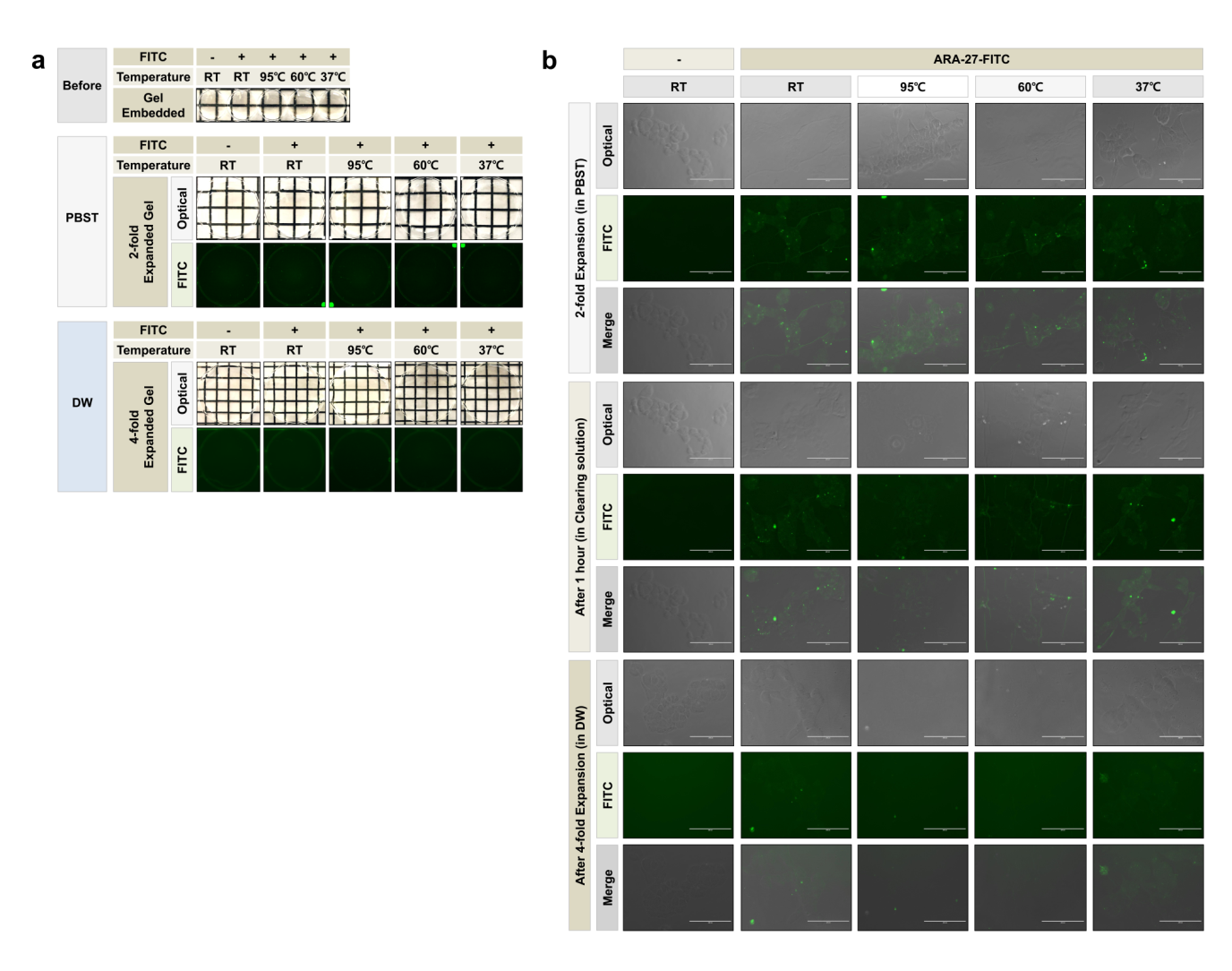** |
| --- |

**Supplementary Figure 5.**

Comparison of (a) hybrid gels and (b) fluorescence preservation efficiency of the Cell-MAP process at different temperature conditions (RT, 95°C, 60°C and 37°C) using Ara-27-FITC transducted 293T cells. The transparency of the transparent Cell-MAP gels was evident against a patterned background (length:width=5 mm:5 mm). All figures were captured with 20x magnification air lenses. Scale bar, 200 μm (white).

| **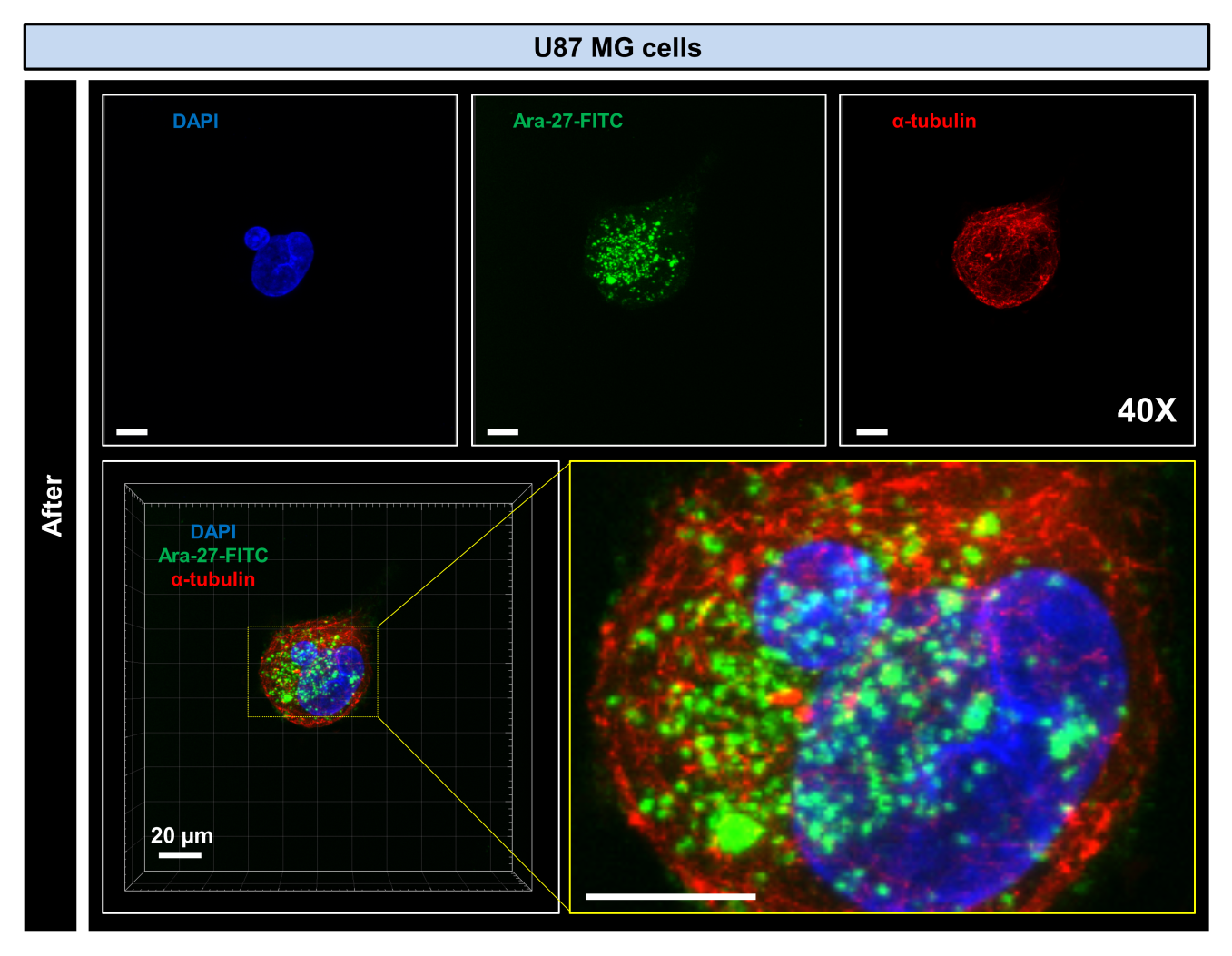** |
| --- |

**Supplementary Figure 6.**

Ara-27-FITC treated U87MG (human primary glioblastoma cell line) cells stained for DAPI and alpha-tubulin, and imaged after Cell-MAP processing. Three-dimensional rendering and focused image (yellow square) of DAPI and alpha-tubulin images after Cell-MAP preparation in Ara-27-FITC peptide treated U87MG cells. DAPI was used to label nucleic acids. DAPI (blue), Ara-27-FITC (green), alpha-tubulin (red). Scale bars, 20 μm (white).

**Supplementary Video Legends**

**Supplementary Video 1.**

Visualization of α-tubulin and nucleus in hybrid expanded 293T cells and morphology using Cell-MAP.

**Supplementary Video 2.**

Visualization of Ara-27-FITC peptide, α-tubulin and nucleus in 293T cells of after Cell-MAP.

**Supplementary Video 3.**

Visualization of Ara-27-ISP-FITC peptide, mitochondria and nucleus in 293T cells of after Cell-MAP.

**Supplementary Video 4.**

Visualization of Ara-27-ISP-FITC peptide, golgi bodies and nuclei in 293T cells after Cell-MAP.
