# Supplementary material for: A Modified Magnified Analysis of Proteome (MAP) Method for Super-Resolution Cell Imaging that Retains Fluorescence
Source: Sci Rep. 2020 Mar 6;10:4186. doi: 10.1038/s41598-020-61156-2 (PMC7060248; doi:10.1038/s41598-020-61156-2)
Supplement: Supplementary file 6 — Check list for submissions. [file 41598_2020_61156_MOESM6_ESM.pdf]

Checklist for initial submissions

SCIENTIFIC REPORTS

Your manuscript will be subject to a quality check before it can be presented to an Editorial Board Member (EBM) for assessment. This is to ensure that all of the information required by the EBM and peer-reviewers is available.

Please review the checklist before submitting, as manuscripts that do not comply may be returned for changes before they are assigned to an EBM. Additionally, please ensure that all author information entered both in your manuscript and on our system is accurate.

Should you have any questions regarding this checklist, please contact us at scirep.admin@nature.com

**Submission Files**			
**Item**	**Permissible file format**	**Notes**	**Completed**
Cover letter	.doc, .docx, .pdf	Will be seen by the EBM but not the peer-reviewers	☑
Article File	.doc, .docx, .tex, .pdf	Only one file permitted	☑
Main Figure File(s)	.jpg, .eps, .tiff, .psd, .png, .pdf	Can be uploaded separately or included within the Article file	☑
Main Table(s)	Do not upload separately	Must be included in the Article File	☑
Supplementary Information	.txt, .gif, .html, .doc, .jpg, .swf, .mov, .xlsx, .pdf, .ppt, .wav, .csv, .zip	Can be uploaded separately or included within the Article file. Supplementary datasets should be provided as separate .xlsx files and uploaded as 'Supplementary Dataset' files.	☑

**Article File**	**Completed**
**TITLE**	
Exactly matches the title entered on the submission system and in any supplementary information	☑
**AUTHORS**	
A maximum of three corresponding author(s) are identified using an asterisk	☑
All author (corresponding and contributing) details are included on the submission system as well as in the Article File	☑
Affiliations are provided and linked to authors with superscript numbers	☑
The individual contribution of each author is specified in the author contributions statement	☑
For papers with consortia as part of the authorship, please include the consortium name as a main author in the author list on the title page. Please provide a full list of members of this consortium at the end of your Article file, after the references, providing affiliations marked by superscript numbers as per the main author list. The consortium name should also be entered as an author on the submission system, together with the contact details of a nominated consortia representative.	☑
**ABSTRACT**	
Exactly matches the abstract entered on the submission system	☑
Does not contain citations	☑
**MAIN TEXT**	
No footnotes	☑
**METHODS**	
Included in main Article File and contain sufficient detail to repeat experiments	☑
For experiments involving live vertebrates and/or higher invertebrates, the Methods section must include a statement that both: (i) identifies the institutional and/or licensing committee that approved the experiments, including any relevant details; and (ii) confirms that all experiments were performed in accordance with relevant named guidelines and regulations	☑

For experiments involving human subjects (or tissue samples) the Methods section must include a statement that: (i) identifies the institutional and/or licensing committee that approved the experiments, including any relevant details; (ii) confirms that all experiments were performed in accordance with relevant named guidelines and regulations; and (iii) confirms that informed consent was obtained from all participants and/or their legal guardians	☑
Please note that: (i) study participant names (and other personally identifiable information) must be removed from all text/figures/tables/images; (ii) the use of coloured bars/shapes or blurring to obscure the eyes/facial region of study participants is not an acceptable means of anonymisation. For manuscripts that include information or images that could lead to identification of a study participant, the Methods section must include a statement that confirms informed consent was obtained to publish the information/image(s) in an online open-access publication.	☑
Scientific Reports will not consider manuscripts describing research that involves organs/tissues procured from prisoners. Authors of manuscripts describing human transplantation research must include a statement in their manuscript attesting that no organs/tissues were procured from prisoners. Authors must also name all institution(s)/clinic(s)/department(s) via which organs/tissues were procured	☑
**AUTHOR CONTRIBUTIONS STATEMENT**	
Author contributions statement, specifying the individual contribution of each author, is provided. This should be included after the references, and every author's contribution must be listed	☑
**ADDITIONAL INFORMATION**	
Competing interests statement is provided in the Article File under the heading "Additional Information", and matches the information entered on the submission system. Competing financial and non-financial interests should be disclosed	☑
**LEGENDS**	
A legend is provided for each main table and main figure in the Article file. These should be placed at the end of the manuscript, after the references	☑
**REFERENCES**	
In a numbered list and all in-line citations should be matched back to a reference via numbering in superscript	☑
**MAIN FIGURES**	
As an online journal, Graphical abstracts, 'Cover' images or table of contents figures are not permitted and must be labelled and numbered as either a figure or table as appropriate.	☑
Please ensure you have read our digital image integrity and standards policy	☑
Gel and blot images are presented in compliance with our digital image and integrity polices. Where cropped gels/blots are displayed, this should be noted in the figure legend; full-length/uncropped gels and blots should be included in a Supplementary Information file	☑
**MAIN TABLES**	
Main tables are provided in the Article File, and not as separate files, in an editable format (not embedded as an image in the document)	☑
